# Exercise Stress Echocardiography for Stable Coronary Artery Disease: Succumbed to the Modern Conceptual Revolution or Still Alive and Kicking?

**DOI:** 10.31083/j.rcm2308275

**Published:** 2022-07-26

**Authors:** Andrea Barbieri, Francesca Bursi, Gloria Santangelo, Francesca Mantovani

**Affiliations:** ^1^Division of Cardiology, Department of Diagnostics, Clinical and Public Health Medicine, Policlinico University Hospital of Modena, University of Modena and Reggio Emilia, 41125 Modena, Italy; ^2^Division of Cardiology, Heart and Lung Department, San Paolo Hospital, ASST Santi Paolo and Carlo, 20122 Milan, Italy; ^3^Department of Health Sciences, University of Milan, 20122 Milan, Italy; ^4^Division of Cardiology, Azienda USL–IRCCS di Reggio Emilia, 42122 Reggio Emilia, Italy

**Keywords:** exercise stress echocardiography, coronary artery disease, functional tests

## Abstract

The modern conceptual revolution in managing patients with stable coronary artery disease (CAD), based on improvement in preventive and pharmacological therapy, advocates coronary artery revascularization only for smaller group of patients with refractory angina, poor left ventricular systolic function, or high-risk coronary anatomy. Therefore, our conventional wisdom about stress testing must be questioned within this new and revolutionary paradigm. Exercise stress echocardiography (ESE) is still a well-known technique for assessing known or suspected stable CAD, it is safe, accessible, and well-tolerated, and there is an widespread evidence base. ESE has been remarkably resilient throughout years of innovation in noninvasive cardiology. Its value is not to be determined over the short portion of diagnostic accuracy but mainly through its prognostic value evident in a wide range of patient subsets. It is coming very close to the modern profile of a leading test that should include, in addition to an essential accettable diagnostic and prognostic accuracy, qualities of low cost, no radiation exposure, and minor environmental traces. In this review, we will discuss advantages, diagnostic accuracy, prognostic value in general and special populations, cost-effectiveness, and changes in referral patterns of ESE in the modern era.

## 1. Introduction

Exercise represents the archetype of stress testing for diagnosing stable coronary artery disease (CAD). The opportunity to evaluate left ventricular wall motion by exercise stress echocardiography (ESE) originated with the use of M-mode echocardiography [1, 2]. The initial landmark report by Wann *et al*. [3] in 1979 documented the value of 2D echocardiography in identifying ESE-induced wall motion abnormalities and their resolution after successful coronary artery bypass surgery. But it was only in the mid-1980s, when early offline digital acquisition systems became accessible, that Armstrong *et al*. [4] showed ESE’s additive and complementary value to standard treadmill parameters when the ECG test was non diagnostic. Since the early 1990s, ESE has become a popular clinical tool, increasingly used for diagnosing, functional assessment, and risk stratification of CAD. However, many things have gradually changed in the meantime. Ten years ago, the 2012 Guideline for the diagnosis and management of patients with stable ischemic heart disease placed more weight on patient-centered care for the first time [5]. Simultaneously, the natural history of patients with stable CAD has also been explained, highlighting the common symptoms resolution over time with a generally good prognosis [6] which challenges the diagnostic evaluation [7, 8]. These developments have been conducted in a new epoch for the evaluation and management of the patient with stable CAD and are well captured in the new 2021 guidelines for the evaluation and diagnosis of chest pain [9] in which detailed recommendations on the use of current models to estimate risk and pretest probability of CAD are recommended. Furthermore, latest guidelines propose the selective use of modern imaging techniques, the specific evaluation of nonobstructive CAD, and listed the aspects to ponder when selecting between coronary computed tomography angiography (CCTA) and stress testing. The modern conceptual revolution in managing patients with stable CAD, based on improvement in preventive and pharmacological therapy corroborated by available robust scientific data from randomized trials, advocates coronary artery revascularization only for smaller group of patients with refractory angina, poor left ventricular systolic function, or high-risk coronary anatomy [6, 10, 11, 12, 13, 14, 15]. Therefore, our conventional wisdom about stress testing must necessarily be questioned within this new and revolutionary paradigm. Accordingly, in this review, we intend to critically update he current role of ESE for the diagnosis and management of stable CAD.

## 2. Advantages of Exercise Stress Echocardiography

Exercise is the most physiologic and familiar stressor. Normally, with exercise coronary blood flow increases up to four-fold [16] and can stimulate myocardial oxygen consumption by up to 4 to 8 times above baseline, mainly through a rise in elastance (i.e., the rise in end-systolic pressure divided for end-systolic volume) [17]. Exercise-induced ischemia is more severe than dobutamine-induced, owing to the higher workloads attained [18]. Echocardiography during physical stress is the only method that combines symptoms’ elucidation, workload, and wall-motion abnormalities and complements echocardiography information with well-established and corroborated electrocardiographic and hemodynamic data. Nevertheless, despite these assumptions, ESE is not considered a routine method for diagnostic and risk assessment of patients with chronic chest pain since it is perceived as a challenging and demanding technique [19]. The introduction of dipyridamole [20] and dobutamine [21] as pharmacological stressors, several laboratories started to use pharmacological stressors even in patients capable to exercise. This is probably the main reason why outcome data are only available on pharmacological stressors from large-scale, multicenter, effectiveness studies [22, 23], suggesting stronger evidence for their use in daily practice. However, it is essential to recognize that nowadays, the digital echocardiographic techniques [24], the enhanced endocardial border detection by harmonic imaging [25], and ultrasound contrast agents that opacify the left ventricle [26] allow diagnostic ESE in many more patients than in the past [27]. ESE is the only non-invasive method that does not necessitate an intravenous line, typically conducted and evaluated quickly by cardiology experts during a single procedure, with the findings usually accessible right after. Additional significant advantages of ESE over other stress imaging modalities include its wide accessibility, portability, low cost since ESE has become widely implemented to assess various conditions other than CAD [28]. Lifestyle changes with ESE results were observed at 2-year and 5-year follow-up [29, 30], suggesting a potential impact of this diagnostic test in primary prevention in women, beyond the immediate diagnostic implications of the test result [29].

## 3. Ethical and Safety Issues

Although patients sent to pharmacological stress may be more commonly “sicker” than patients able to exercise, the existing evidence indicates that ESE is safer than pharmacological stress, with only one major life-threatening adverse event in every 6000 exams, 5-fold less than with dipyridamole echocardiography, and 10-fold less than with dobutamine echocardiography [31]. According to the American Heart Association statements on exercise testing, death occurs on average in 1 in 10,000 tests, grounded on a review of more than 1000 studies on millions of patients [32]. This matter was addressed by many international guidelines, saving drug-induced stress echocardiography only for patients not capable to exercise [33, 34, 35].

**Fig. 1.**
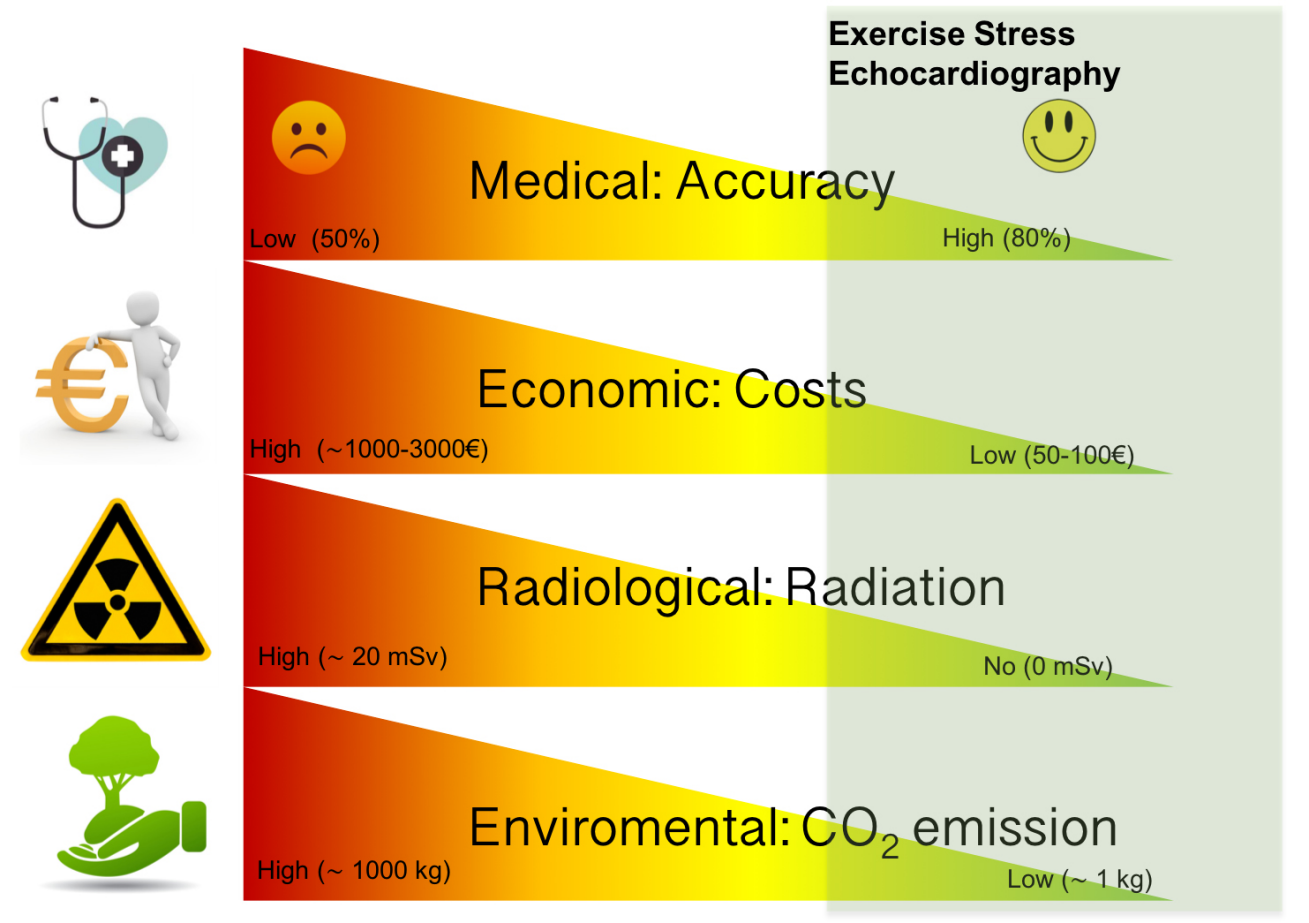
**The dimensions of sustainability in ESE**. Like all imaging modalities, exercise stress echocardiography can be categorized in a system with different dimensions including diagnostic/prognostic accuracy from physician perspective, economic/costs from payer perspective, radiation exposure from patient perspective, environmental footprint, from planet perspective. The green area subtended supports a leading and current role of exercise stress echocardiography in patients with stable coronary artery disease. ${\mathrm{CO}}_{2}$, carbonic oxide; ESE, exercise stress echocardiography.

An additional significant value to consider is that ESE is a green sustainable technology. In imaging rationalization, a clinician should consider the cost/benefit ratio, and the biological risk, including long-term cancer hazard [36] and environmental traces [37], especially if serial studies are needed (Fig. 1).

## 4. Valuable Information Regarding Functional Status 

ESE is beneficial for prognostic assessment because it provides valuable information regarding the functional status and other exercise variables with well-established predictive value, such as metabolic equivalents reached (METs), chronotropic and blood pressure reaction, heart rate reserve, or achieved age-predicted maximal heart rate [38]. Some individuals may show limited exercise competence, as 25%–30% of the patients do not reach 85% of the age-predicted maximal heart rate, therefore the test cannot be considered conclusive [39]. However, it is essential to point out that irrespective of the heart rate reached, exercise capacity measured in METs represents an independent mortality risk predictor, better than angiographic severity of CAD [40, 41]. Appropriate exercise capability is associated with diminished mortality, acute myocardial infarction, and coronary artery revascularization, even in presence of ischemic ECG changes such as ST-segment depression [42, 43].

An exercise capability superior to 10 METs selected an excellent survival group despite the amount of CAD or the presence of left ventricular dysfunction that excludes any survival benefit from coronary artery revascularization [44] with massive consequences for expense containment and medical care.

Capability to exercise is associated to more than just cardiovascular fitness. It depends on a combination of many factors, involving normal lung performance, the health condition of other organs, nutritional status, nitrogen balance, drugs, orthopedic restrictions between others [41]. Despite its vast predictive value, the application of functional capacity in daily clinical practice is challenging for the scarcity of standardization. Functional capacity tends to decline with age and for any given age is different for gender (higher in men than in women). Notwithstanding, measurements of <5 METs are considered the threshold defining functional disability. Through the available literature on functional capacity, the 5 METs-threshold represents a reliable marker of worse prognosis [43, 45].

Current guidelines offer physicians little guidance on identifying patients who would not complete the exercise test sufficiently. Data from the Women’s Ischemia Syndrome Evaluation (WISE) Study group have provided insight into the ideal identification of candidates eligible for ESE versus pharmacologic stress by applying the Duke Activity Status Index (DASI score) before stress testing. Patients presenting for evaluation of CAD with estimated METs <4.7 were better served by pharmacologic stress imaging encounters [46].

On the other hand, the inability to undergo an exercise test, resulting in the decision for a pharmacologic stress test, has been shown in numerous papers to be an independent variable associated with a poor outcome. It is worth noting that individuals who undergo pharmacologic stress testing have a worse prognosis for similar echocardiographic findings than exercise [47].

## 5. Various Methods of Exercise Stress Echocardiography 

Treadmill and semi-supine bicycle ESE are the two commonest ESE modalities. Most laboratories in the United States employ the post-treadmill technique, with imaging performed at rest and as quickly as feasible during early recovery [34]. The treadmill has certain benefits over the bicycle, such as increased O2 consumption. Still, all patients who can exercise can successfully walk on a treadmill. In contrast, issues with pedaling or pausing pedaling is common in unskilled patients on a bicycle. Muscular soreness before reaching the age-predicted submaximal heart rate is another typical cause for terminating bicycle workout [48].

In Europe, on the other hand, numerous institutions have equipped their ESE laboratories with a specific bed or table that allows semi-supine cycling exercise and continuous real-time imaging during the exercise [49].

**Fig. 2.**
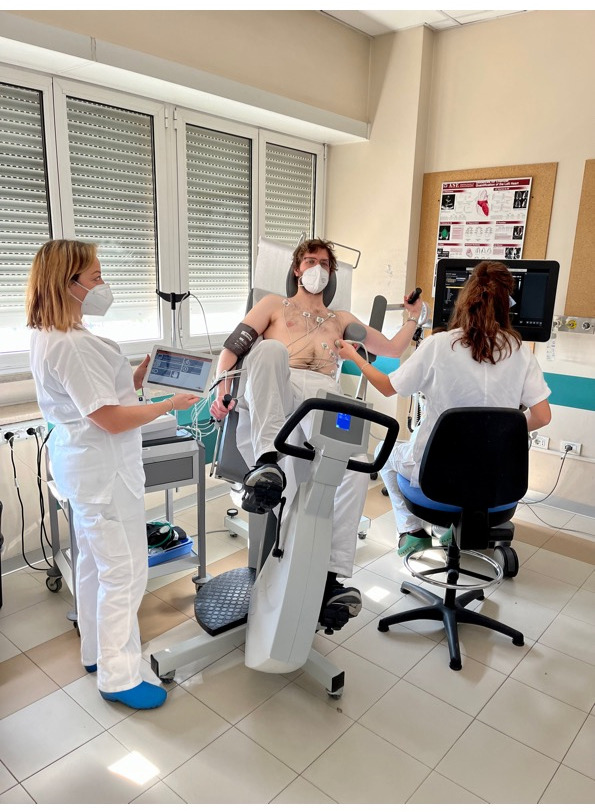
**Echocardiographic data acquisition with semi supine bicycle**. ESE is conducted with a adjustable load supine bicycle ergometer on a reclining seat position to find satisfactory echocardiographic views. A standard exercise protocol is applied with a rise of 25-Watt every two minutes, while the patient sustained a fixed rhythm at 50–60 rotation/minute. Two-dimensional images are acquired in 4-standard views at baseline, at each step and in the recovery phase. During every step of exercise and recovery, any eventual symptoms or arrhythmias are recorded together with blood pressure, heart rate, and 12- lead ECG. ESE, exercise stress echocardiography; ECG, electrocardiogram.

We started performing semi-supine ESE more than 20 years ago because our feeling was of a much more user-friendly exam than the treadmill test, making image acquisition simpler and interpretation quicker, as previously suggested by other laboratories (Fig. 2) [50, 51].

We also considered other fundamental differences from other forms of exercise. Although length of exercise and maximum achieved heart rate are a little worse in the supine position onset of leg weakness at an earlier stage of workout [52, 53] the appearance of ischemia at a lower threshold of workload with supine exercise overcomes this limitation. Indeed, for a given degree of stress in the supine posture, a larger end-diastolic volume and a higher mean arterial blood pressure cause ischemia to occur quicker. Compared to an upright bicycle, these differences promote a more substantial wall stress and increased myocardial oxygen demand [54].

When compared to evaluation limited to the time before and after exercise, the comparative benefit of ESE with image capture during semi-supine activity revealed an enhanced diagnostic accuracy for CAD [51, 55, 56, 57, 58], multivessel CAD [59], and the assessment heart failure, of pulmonary hypertension, valve diseases, cardiomyopathies, which are becoming increasingly used together with CAD assessment [28].

## 6. Diagnostic Accuracy and Prognostic Value

In general, an image-based functional test is more specific than a typical exercise ECG, and the accuracy of the different non-invasive imaging functional tests is comparable [35]. The overall sensitivity and specificity of ESE have been reported to be 83 and 84%, respectively, according to the most updated meta-analysis of 55 studies with 3714 patients. The specificity of ESE is similar to dobutamine echocardiography, lower than dipyridamole echocardiography, and higher compared to stress single-photon emission computed tomography (SPECT) [60].

As with any form of stress testing, the sensitivity for detecting CAD is higher in patients with multivessel disease than in those with single-vessel disease [61] and lower when compared with fractional flow reserve, considered the ‘gold standard’ for diagnosing ischemia-causing hemodynamically significant CAD [62], although all coronary lesions were not automatically identified like any symptom-limited stress test that should be discontinued at the onset of ischemia in the most critical coronary territory and, therefore, not progressing to the point of unmasking less severe stenoses. As with all approved tests in clinical practice, reports of ESE performance may be influenced by referral bias that occurs when patients with an abnormal stress test result are referred to Cath lab at a higher rate than patients with normal stress test. Indeed, after analytic estimates adjustment for referral, ESE sensitivity fell from 84% (80% to 89%) to 34% (27% to 41%), and the specificity rose from 77% (69% to 86%) to 99% (99% to 100%) [63]. Although other critical unmeasured characteristics may affect ESE diagnostic performance (e.g., patient-level risk, the severity of symptoms, and adequacy of the heart rate response), the prognostic value of ESE in terms of adverse cardiovascular events is also cited as a element of its diagnostic utility. Sawada *et al*. [64] in 1990, for the first time, demonstrated an excellent intermediate-term prognosis in patients with a normal ESE. Subsequently, the prognostic value of both positive and negative ESE results has been demonstrated in sizeable observational series with low rates of CAD events for patients with normal test results, particularly those with good exercise tolerance, both in the general population and in specific patient subsets [65, 66, 67]. Arruda-Olson *et al*. [68] demonstrated a slightly higher event rate in men than in women, but a statistically significant correlation between wall-motion score index at stress and the likelihood of adverse outcomes during follow-up. A meta-analysis of studies published between 1990 and 2005 found that a normal ESE (defined as normal wall motion at rest and with stress) had a 98.4% negative predictive value for the hard endpoints of myocardial infarction and cardiac mortality during a 33-month follow-up with no difference between male and female [69]. As previously mentioned, the inability to exercise is by itself an ominous prognostic sign. Consequently, patients referred for pharmacological stress echocardiography have a higher event rate than those referred for ESE. Chaowalit *et al*. [70] demonstrated that the outcome after normal dobutamine stress echocardiography is not as good as that reported after normal ESE. In the context of inducible wall motion abnormalities, ESE characteristics such as ischemic threshold and the amount and severity of ischemia affect the probability of developing unfavorable outcomes. Peteiro *et al*. [71] showed that ESE could further differentiate patients with an intermediate Duke treadmill score into those at higher and lower risk of events and has incremental predictive value in patients with different pre-test probabilities of CAD [71]. ESE has shown a substantial clinical relevancy in ischemia detection because of its high sensitivity and specificity both in patients without known CAD [72] and in those previously undergone to percutaneous coronary intervention [73] or coronary artery bypass graft surgery [74]. In the setting of an abnormal ESE electrocardiogram, the evidence is still debated. Our group and others [75] showed an excellent long-term prognostic value of negative ESE regardless of electrocardiogram results. Conversely, a large observational study conducted at Duke University Medical Center in 15,077 patients without known CAD who underwent ESE showed that the presence of exercise-induced ST depression with normal ESE imaging might identify a subset of patients who are at slightly increased risk for adverse cardiac events after a median follow-up of 7.3 years [76]. Also the 5 years outcome of the SMART Study (Prognostic Utility of Stress Testing and Cardiac Biomarkers in Menopausal Women at Low to Intermediate Risk for Coronary ARTery Disease) conducted on 400 peri/postmenopausal women undergoing contrast stress echocardiography (almost 80% ESE) showed that both abnormal stress electrocardiogram and abnormal stress echocardiography were associated with cardiac events while only abnormal stress electrocardiogram was an independent predictor of cardiac event within 5 years [77]. However, further studies are needed to determine whether these patients will benefit from the intensification of medical management. In patients with known or suspected CAD, unexplained dyspnea is a symptom requiring investigation, considering that they have a high likelihood of ischemia and an increased incidence of cardiac events [78, 79]. Compared with other modalities of stress testing and noninvasive cardiac imaging, ESE provides independent information for identifying patients at risk offering the plus that other hypothetical cardiac etiologies of dyspnea can also be evaluated at the time of testing [80].

## 7. Special Populations 

Aside its diagnostic utility, the prognostic value of ESE has been demonstrated in a variety of patient populations, including subjects ≥65 years of age [81], women [77, 82, 83, 84, 85], patients with LV hypertrophy [86, 87], left bundle branch block [88, 89], atrial fibrillation [90], diabetes mellitus [91, 92], heart transplant recipients [93, 94], and candidates for renal transplantation [95]. The prognostic value of ESE in each of these selected groups (Table 1, Ref. [77, 78, 79, 80, 81, 82, 83, 84, 85, 86, 87, 88, 89]) is supported by robust evidence that corroborates its use in clinical practice.

**Table 1.** **Studies of the prognostic value of Exercise Stress Echocardiography in special population**.

Reference	Special population	N	Mean follow-up	Mean age, yrs	Event rate after a negative ESE, %	Negative predictive value, %	Event rate/years, %
					(95% CI)	(95% CI)	
Arruda *et al*. [77]	Age >65 years	2632	2.9 ± 1.7 years	72 ± 5	NA	NA	1.9%/year (cardiac death and non-fatal myocardial infarction)
Marwick *et al*. [78]	100% women	161	NA (cross-sectional design)	60 ± 8	NA	87% for CAD	NA
Deng *et al*. [79]	60% women	30 cases with mild stenosis	NA (case-control design)	68.80 ± 3.93	NA	NA	NA
		30 controls					
Sawada *et al*. [80]	100% women	57	NA, angiogram within 3 weeks	57 (range 33 to 75)	Significant CAD: 23.5%/year treadmill; 0%/year bicycle	91% treadmill; 100% bicycle	16.3/year (significant CAD)
Williams *et al*. [81]	100% women	70	NA (cross-sectional design)	60 ± 9	11.4%	88%	NA
Bangalore *et al*. [82]	LV hypertrophy (677 dobutamine 325 ESE)	1002	2.6 ± 1.1 years	62 ± 13	4.5%/year (total mortality)	88% years (total mortality)	16.3 (total mortality)
					1.1%/years (hard events)	97% years (hard events)	7 (hard events)
Marwick *et al*. [83]	LV hypertrophy	147 (68 with LV hypertrophy)	NA (cross-sectional design)	58 ± 12	NA	NA	41% of LVH atients had significant CAD
Xu [84]	Left Bundle Branch Block	191*	NA (cross-sectional design)	65 ± 11	2.4% (significant CAD)	97.5%	NA
Peteiro [85]	Left Bundle Branch Block (17 with CAD 18 without CAD)	35	NA (cross-sectional design)	66 ± 6 (CAD)	21	79% (68–90) WM abnormalities	NA
				61 ± 8 (no CAD)			
Bouzas-Mosquera *et al*. [86]	Atrial fibrillation	17100	6.5 ± 3.9 years	64.3 ± 8.2 (total)	NA	NA	Mortality 43% in AF in 10 years (TDS)
		619 Atrial fibrillation		69.2 ± 7.6 (Atrial fibrillation)			
		exercise electrocardiography or ESE					
Garrido *et al*. [87]	Diabetes mellitus	214	44 ± 16 months	64 ± 8	1.6%/year	46.7%	4.65%
Elhendy *et al*. [88]	Diabetes mellitus	563	median 2.5 years	64 ± 11	1.3%/year	42%	3.6%
Gebska *et al*. [89]	Heart transplant Recipients	81 (45 ESE)	NA (cross-sectional design)	47 ± 10	2.3% total (not annualized)	100% (death in ESE)	6.66% total (not annualized)

LV, Left Ventricular; LVH, left ventricular hypertrophy; ESE, exercise stress echocardiography; CAD, coronary artery disease; N/A, not available or not applicable; WM, wall motion. * 9 have an inconclusive study.

## 8. Limitations

ESE tends to aggravate the two main “classical” disadvantages of stress echocardiography: the dependency on the acoustic window and the reader’s expertise. ESE is unquestionably more technically challenging and requires more skills than pharmacologic stress due to increased heart and respiratory rates with exercise and a shorter time frame [16, 96]. A poor acoustic window in some patients is the fundamental drawback of ESE. This situation is not uncommon, especially among the elderly, since one out of every five patients referred for ESE has an interpretable but difficult echocardiogram, making pharmacological stress echocardiography a more realistic alternative [49]. However, with the development of harmonic imaging, this number has dropped considerably, and contrast agents can be employed to enhance myocardial boundary delineation [97]. A drawback of any symptom-limited stress test is that it may be terminated at the onset of ischemia in the most critical coronary area, preventing it to reveal more severe stenosis [98]. As a result, the question becomes what we should do in clinical practice to rule out left main CAD before starting a patient with moderate to severe ischemia on medical therapy. A comprehensive examination of a patient’s risk factor profile and noninvasive imaging results can assist in advise, but for now, anatomical imaging is the modality of choice for reliably ruling out left main CAD [98]. There are still limitations due to a relatively subjective interpretation which has led to only moderate agreement between observers in different studies [99] and between site and core laboratory [100] although the agreement is higher when significantly induced wall motion abnormalities are present [101]. In summary, there is no question that the technical difficulties of conducting ESE are fewer during pharmacologic stress, and considerable thought has been given to replacing the former entirely, even in patients who can exercise. However, considering the ratio of benefits and limits, according to many current guidelines [33, 34, 35], ESE must be viewed as the first choice instead of pharmaceutical stressors in patients who can exercise unless a particular benefit of pharmacologic stress is indicated [102].

## 9. Supplementary Echocardiographic Techniques 

**Fig. 3.**
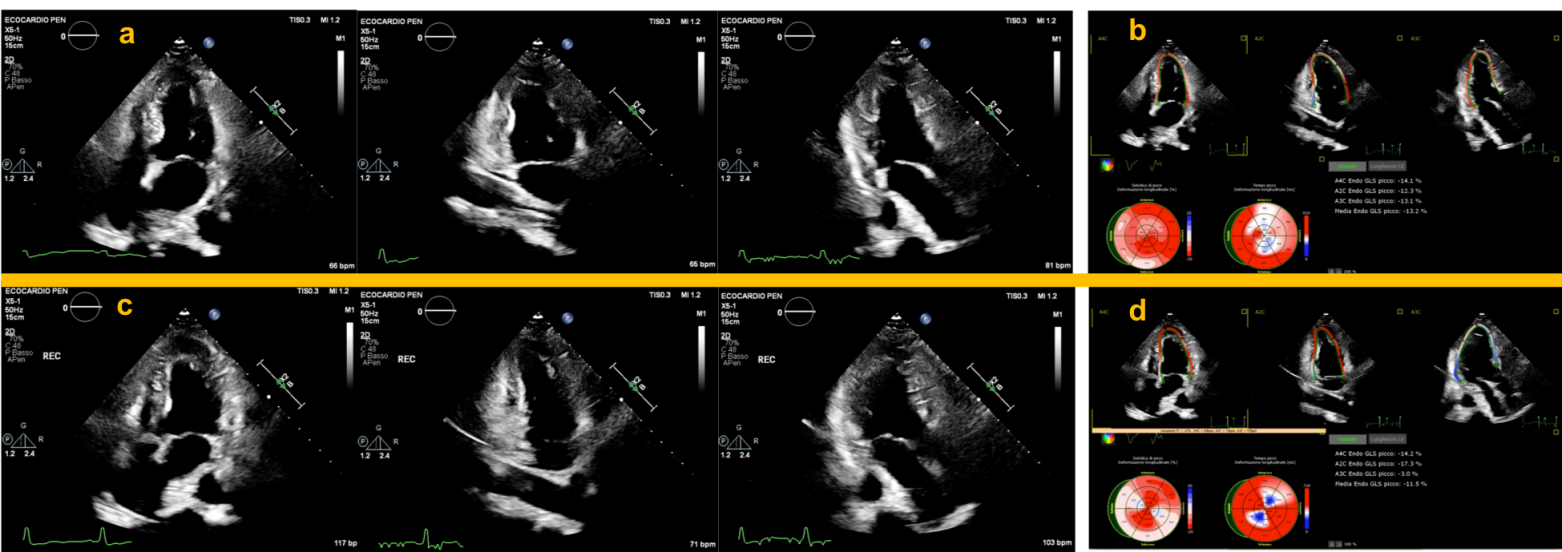
**Application of strain analysis during ESE in patients with severe obstruction of the LAD and RCA**. Upper panels show normal resting apical chambers view during end-systole (a), and strain analysis performed simultaneously, showing a significant reduction in the septal and inferior segments (b). Lower panels: immediate post-exercise apical chambers view depicting a normal WM response with decreased LVESV (c). Strain analysis performed immediately post-exercise showed a well-defined segmental worsening in the LAD and RCA territory compared to baseline values (d). Also note the significant decrease in GLS which points to an increased risk of multivessel coronary heart disease. ESE, exercise stress echocardiography; LAD, left anterior descending coronary artery; RCA, right coronary artery; WM, left ventricular wall motion; LVESV, left ventricular end-systolic volume; GLS, global left ventricular longitudinal strain.

Given its constraints due to suboptimal image quality and decreased endocardial border detection, ESE may become the ideal arena for additional technology in different ways: more quantitative assessment of the regional wall thickening, endocardial border delineation, myocardial perfusion by contrast-enhanced imaging, and evaluation of coronary flow reserve. However, none of these technologies currently has a place in the routine clinical practice of ESE. Shimoni *et al*. [103] have demonstrated the feasibility and specificity of real-time imaging using qualitative contrast-ESE, but it has low sensitivity for detecting moderate or severe perfusion defects compared with single-photon emission computed tomography. Furthermore, the assessment of myocardial perfusion during ESE intravenous line is not without certain technical limitations producing artifacts (pseudo defects, blooming, myocardial heterogeneity) [104]. While 3D imaging overcomes some of the limits of 2D imaging, it is still constrained by spatial and temporal resolution, particularly during ESE. Continued technical advancements (single beat acquisition, smaller footprint matrix transducers, wider sector angles, and higher frame rates) will increase the diagnostic potential of 3D-ESE as a tool for evaluating suspected CAD [105]. The clinical significance of the ESE-strain analysis has only recently been analyzed and is still in process. Myocardial deformation imaging is a valuable technique in detecting patients with obstructive CAD, especially if conventional ESE is doubtful. Global strain values are significantly correlated with CAD severity [106] and could discriminate left ventricular regional systolic function abnormality sensitively (Fig. 3) even in patients with mild single vessel coronary artery stenosis [83]. Contrariwise, absolute peak GLS ≥20% during ESE excludes obstructive CAD on CCTA [105]. Speckle imaging may also be used to evaluate tardokinesis, which is difficult to observe visually [107, 108]. Contractile reserve measured by myocardial work is reduced in functionally significant CAD, especially in advanced multivessel disease [109, 110, 111]. However, the physical and methodological limitations of the technique boosted during ESE (selection of the velocity settings, gain dependence, angle of the ultrasound beam) should be considered. The combination of anatomical and functional “hybrid” imaging is appealing and provides a new frontier in ESE. Recent developments in the integration of different ESE parameters into a “quadruple protocol” (coronary velo city flow reserve, regional wall motion abnormalities, left ventricular contractile reserve, and stress-induced B-lines) [112, 113, 114, 115] allow for potential incremental prognostication, identifies patients who may benefit from secondary prevention, and improves diagnostic accuracy and risk stratification [116]. However, although coronary flow velocity reserve in the left anterior descending coronary can be obtained during all forms of stress echocardiography with overall feasibility of 80% for ESE in the largescale, international, observational Stress Echo 2020 [117], the experience is limited mainly to vasodilator stress echocardiography. Indeed, the choice of ESE is related to the eightfold risk increase in the loss of left anterior descending coronary flow recorded during peak stress, reflecting hyperventilation and motion of the patients during peak exercise [118]. Also, Doppler quality varies considerably between echo systems. The feasibility data with ESE usually arises from a few centers with a longstanding interest in coronary flow reserve velocity assessment with transthoracic echocardiography. The lack of a standardized protocol for the concurrent use of contrast agents and sufficient training is likely to be the major influencing factor in the more widespread use of this technique. The combination with atherosclerosis imaging by intima-media thickness and/or plaques on carotid ultrasonography and ischemia testing by ESE may lead to a reclassification of the pre-test probability of CAD [119] and have synergistic prognostic value. Major adverse cardiac event rate/year increased from 0.9% in patients with no plaque and normal ESE to 1.95% in the presence of plaque and normal ESE to 4.23% in those with no plaque and abnormal ESE, to 9.58% in those with a plaque and abnormal SE, respectively (*p *< 0.0001) [120]. Artificial intelligence offers a tremendous opportunity to significantly enhance the usefulness of stress echocardiography by increasing its efficiency and reproducibility [121]. Upton *et al*. [122] recently documented for the first time the important role of artificial intelligence-based methodology in improving the accuracy, confidence, and reproducibility of stress echocardiography interpretation in a data set of 578 patients undergoing both dobutamine and ESE, performed using various ultrasound systems, with or without ultrasound image–enhancing agents. The supervised machine learning classifier considered 31 from ~7000 features in model development, including unique geometric and kinematic markers of regional wall motion abnormality and endocardial velocity. The area under the receiver-operating characteristic curve was 0.934, with a sensitivity of 86.7% and specificity of 85.7% for the diagnosis of severe CAD [122]. Notably, when readers used artificial intelligence-based classification to assist in their interpretation, sensitivity increased from 85.0% to 95.0%, without a loss in specificity (from 83.6%–85.0%). Moreover, confidence in interpretation and agreement between readers improved when results from the artificial intelligence-based classifier were considered [122].

## 10. Contemporary Change in the Referral Pattern 

As the patients we send for stress testing evolve, so must our interpretation of the data. Indeed, we found a gradual decline in the frequency of inducible myocardial ischemia in patients with previous or suspected CAD sent to our Echo Lab for ESE over 12 years. This trend was paralleled by changes in ESE referral practice. It is worth noting that the sporadic occurrence of abnormal ESE test findings happened in a cohort with a low-to-moderate pretest risk of CAD during the research period [123]. Although a complete application of appropriateness guidelines is expected to increase the diagnostic yield of the test [124, 125], changes in referral practices are undoubtedly occurring, resulting in an “epidemiological shift” defined by acceptance of patients with a low pre-test risk of CAD, on anti-ischemic therapy, with atypical symptoms, and a previous uncertain exercise ECG [106]. Therefore, noninvasive testing can rarely rule in CAD in a contemporary population with a low disease prevalence, and the focus should shift to ruling-out obstructive CAD [126]. A recent systematic review suggests that for patients with a low-to-intermediate pretest probability, CCTA may be cost-effective as an initial diagnostic imaging test compared with invasive coronary angiography or other non-invasive diagnostic tests. Functional testing represents a cost-effective first strategy only in patients with an intermediate pre-test probability of CAD. Immediate coronary angiography is suggested to be a cost-effective strategy only for patients with a high probability of having obstructive CAD who may profit from coronary revascularization [127].

## 11. Cost/Effectiveness

As healthcare costs rise, better effective methods to diagnose and treat stable CAD are needed. In this regard, the recent PROMISE [15] and SCOT-HEART [128] have opened the path for more significant standards in cardiovascular imaging outcomes research. Currently, worries regarding cost-effectiveness are the only reasons not to switch exercise ECG with ESE. Nevertheless, the first randomized study recently demonstrated that ESE is more efficacious with superior cost-saving than exercise ECG when used as the initial investigation in patients with new-onset suspected stable chest pain, low-intermediate pre-test probability, and without known CAD. Importantly, in this study, inconclusive results after exercise ECG were more than 1/3 compared to only 0.5% in patients who underwent ESE [67]. Therefore, downstream costs are particularly low in patients deemed at low risk by ESE, in contrast with patients estimated at low risk by exercise ECG results.

## 12. The Competitors

Imagers must be familiar with the strengths and weaknesses of various imaging methods to guarantee the optimal selection of the best test. Regarding its accuracy for detecting patients with stable CAD, ESE has been compared directly and indirectly to competing methods. Many investigations have shown similar accuracy, with radionuclide-based perfusion imaging modalities having slightly greater sensitivity and echocardiographic imaging having a slightly higher specificity [129]. Magnetic resonance may compete with ESE in the future because it is also a radiation-free technology with better sensitivity [126]. However, it is still not sufficiently available [130], and most protocols are centered on pharmacological stressors rather than exercise. CCTA has emerged as the real noninvasive competitor based on five randomized, controlled trials conducted over the past 10 years [131, 132]. The 2016 update of the National Institute for Health and Care Excellence (NICE) guidelines for the management of chest pain of recent onset [133] and the 2019 European Society of Cardiology for the diagnosis and management of chronic coronary syndromes [134] have significantly contributed to a shift in practice, elevating CCTA to class I indication as the initial test to diagnose CAD, equaling the strength provided to stress imaging. The more recent guidelines recognize the prevalence and importance of ischemia and no obstructive CAD for the first time and rely on new evidence to elevate the use of anatomic testing while acknowledging the long-term usefulness of stress imaging [35, 135]. Although it suffers from reduced specificity among patients with intermediate stenosis [62, 136], CCTA is the only non-invasive test that can qualitatively and quantitatively assess specific features defined as ‘adverse plaque’ phenotype [137, 138, 139, 140]. A recent survey showed that in patients presenting for the first time with chest pain, 1/3 of centers move directly to CCTA and 15% chose stress echocardiography. Conversely, in patients with established CAD and recurrent chest pain, stress echocardiography and nuclear stress perfusion scans were the preferred tests for decision making [130].

## 13. Selecting Appropriate Testing and the “Patients-First” Cardiac Imaging Approach

**Fig. 4.**
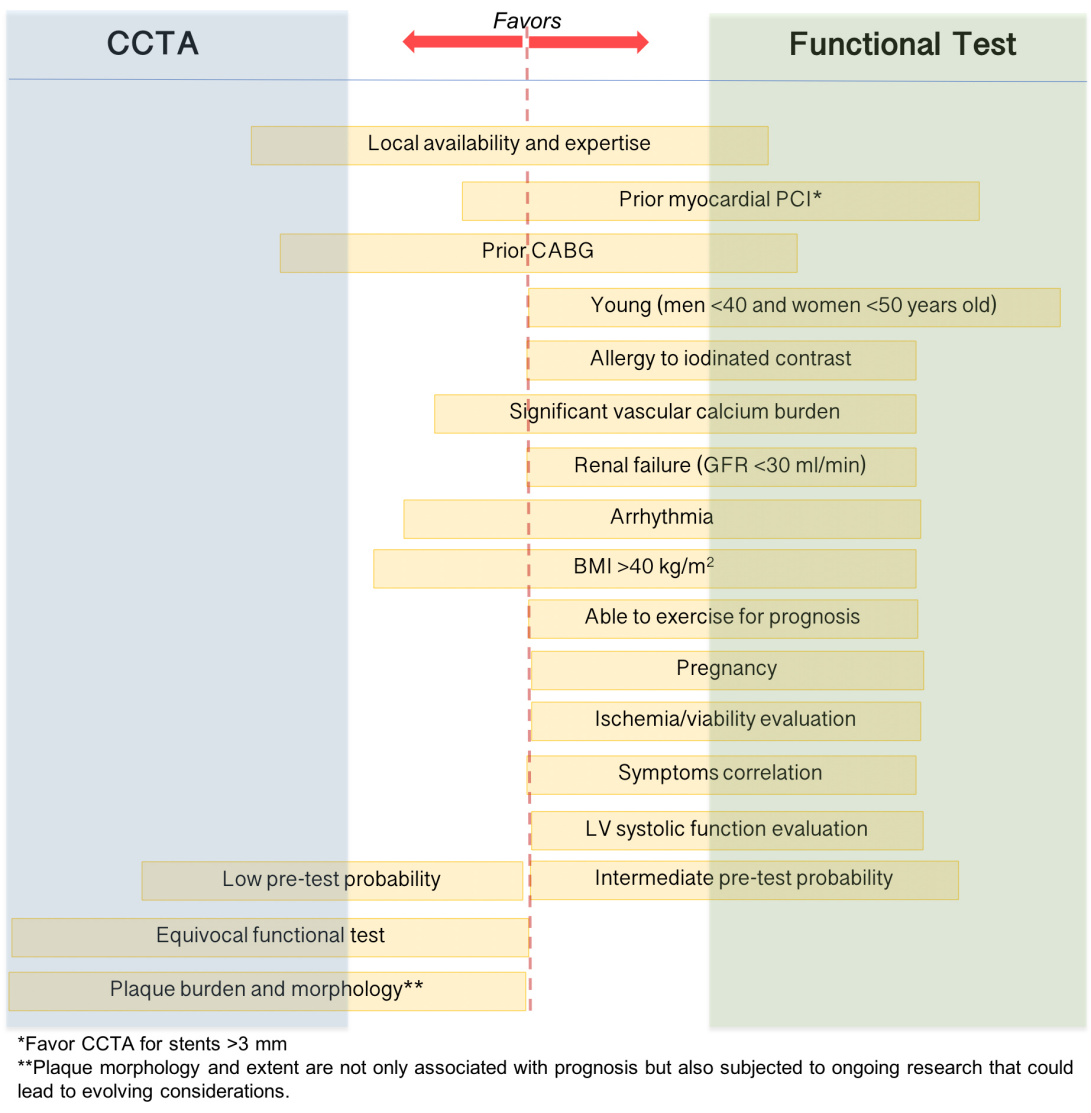
**Patient first strategy for stable coronary artery disease evaluation**. Only the main factors that influence the final decision are mentioned. The length and direction toward CTTA or functional test of the yellow box enlisting variables illustrated the “weight” of each variable in the decision whether to perform a functional or anatomical noninvasive test. CCTA, coronary computed tomography angiography; PCI, percutaneous coronary intervention, CABG, coronary artery bypass graft; GFR, glomerular filtration rate in ml/min/1.73 $m^{2}$; BMI, body mass index; LV, left ventricle.

**Fig. 5.**
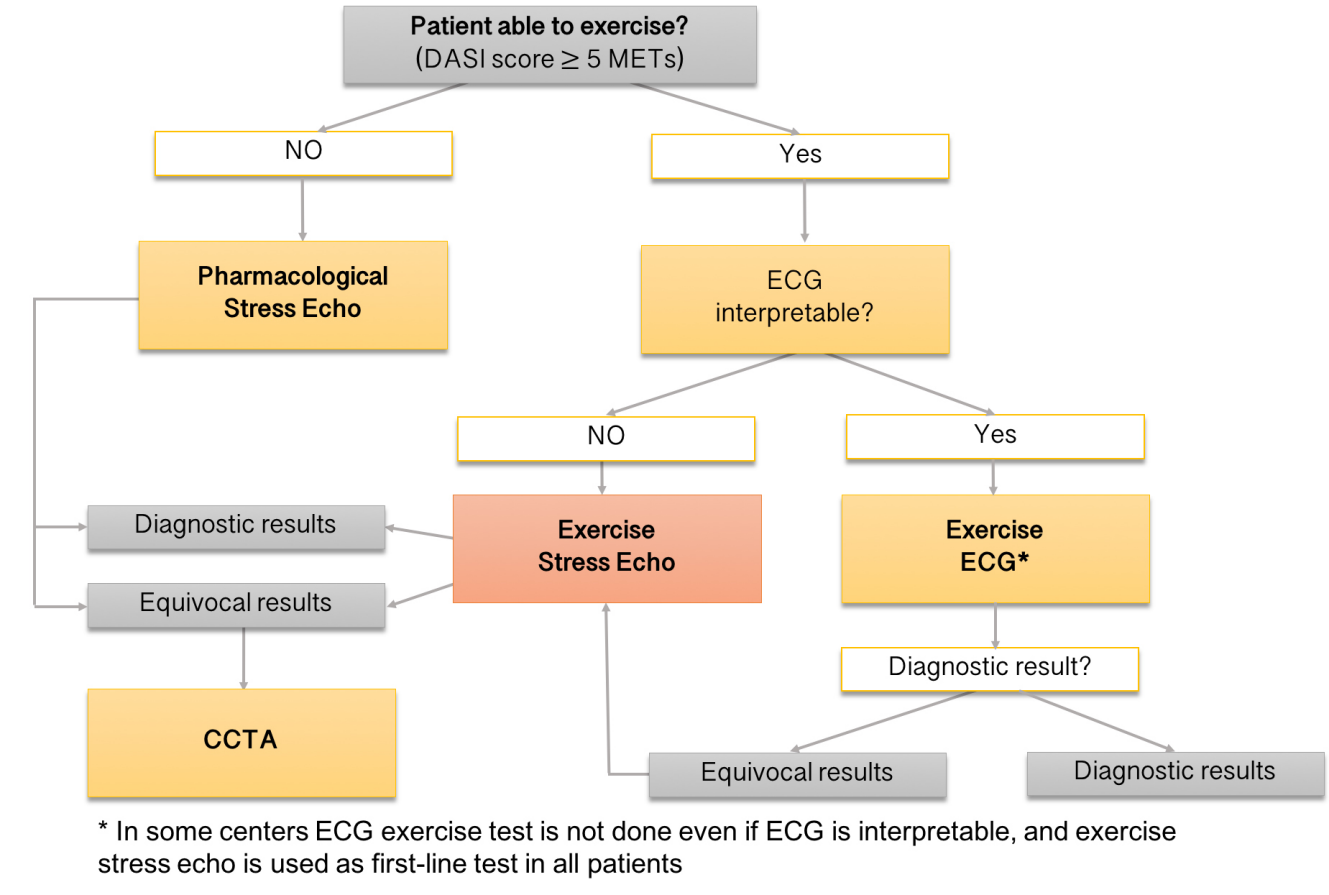
**The suggested algorithm for the use of ESE**. In our institution, subjects with resting ECG changes, known CAD, unexplained exertional dyspnea, or intermediate pre-test probability of CAD are preferentially referred for ESE. ESE is also the most appropriate second-line stress test after inconclusive exercise ECG. The diagnostic test option considers our site-specific availability. CAD, coronary artery disease; DASI, Duke activity status index; METs, metabolic equivalents; ECG, electrocardiogram; ESE, exercise stress echocardiography. CCTA, coronary computed tomography angiography.

While the recommendations acknowledge that all modalities may be acceptable for testing for stable CAD, they now offer some advice on which tests to use depending on clinical characteristics. In general, if a clinician aims to rule out CAD, higher sensitivity of a noninvasive test, such as CCTA or other novel technologies, may be more correct tests to use initially. If a clinician aims to rule in CAD in the same setting instead, stress imaging may be considered the more proper test according to its high specificity. However, numerous factors influence the final shared decision, to mention only a few, the local expertise, test availability, individual contraindications to exercise or pharmacological stress testing, concurrent indications for thoracic imaging, suspected structural heart abnormalities, and not least, the individual sensitivity to the issue of sustainability and patient preference (Fig. 4) [141]. Furthermore, the delivery of CCTA services varies greatly between health areas and even throughout developed nations with cardiological and radiological multi-disciplinary reporting performed only in around a quarter of centers [134], necessitating major investment in new technology, training, and expertise to support the spread of high-quality CCTA [142]. Therefore, test selection via a philosophy of the “right test for the right patient” for the specific setting has become an integral part of clinical practice. While we do not suggest the formula that “further clinical trials are required”, we do stress the significance of rigorous clinical evaluation of patients and a pragmatic, patient-centered approach to CAD testing (Fig. 4) [143]. From a practical standpoint, in our institutions, a systematic replacement of exercise ECG with ESE could not yet be feasible logistically, and appropriate selection is required. Patients with either resting ECG changes, previous CAD, unexplained exertional dyspnea, or intermediate pre-test probability of CAD are better referred for ESE. ESE is also the most suitable second-line stress test when exercise ECG, performed as a first-line test, reproduced ST-segment depression without angina or when the positive predictive value of these findings remains low (e.g., in women and/or hypertensive subjects). In our practice, patients with normal ESE (defined as normal wall motion at rest and with stress) with or without known CAD represent a low-risk population requiring no further imaging. Conversely, after an inconclusive ESE, patients with intermediate-high risk are sent to CCTA (Fig. 5).

## 14. Conclusions

ESE is still an established technique for assessing known or suspected stable coronary artery disease (CAD). It is recommended by all cardiology guidelines in several clinical settings, and we expect that its role will remain central for a long time. It is safe, accessible, and well-tolerated, and there is a large data evidence-based documenting its clinical value. ESE has been remarkably resilient throughout years of innovation in noninvasive cardiology, offering cardiac reassurance to most chest pain patients with no or minimal ischemia across a wide range of symptoms and pre-test likelihood of disease. The value of ESE is not to be measured over the short segment of diagnostic accuracy, but mainly through its prognostic value evident in a broad range of patient subsets. This represents the most beneficial clinical feature for modern cardiology if we consider the revolutionary new paradigm where clinicians should apply the initial test results mainly to intensify guideline-directed medical therapies and direct the need for follow-up testing, reserving invasive angiography for patients who have high-risk anatomy or refractory symptoms. It is coming very close to the modern profile of a leading test that should include, in addition to an essential adequate diagnostic and prognostic accuracy, features of low cost, trivial radiation exposure, and minimal environmental traces. When the cost-effectiveness of emerging procedures is being investigated, we feel a better consideration could be: is their widespread performance and reimbursement justifiable? The current shift toward using ESE protocols with both known and supplementary echocardiographic techniques is a new frontier. It will be fascinating to see how additional technology, such as the use of artificial intelligence in ESE, will affect our practice in the coming years.
